# Identification of a *nth*-Like Gene Encoding an Endonuclease III in *Campylobacter jejuni*

**DOI:** 10.3389/fmicb.2019.00698

**Published:** 2019-04-10

**Authors:** Lei Dai, Jing Xia, Orhan Sahin, Qijing Zhang

**Affiliations:** ^1^Departments of Veterinary Microbiology and Preventive Medicine, College of Veterinary Medicine, Iowa State University, Ames, IA, United States; ^2^National Risk Assessment Laboratory for Antimicrobial Resistance of Animal Original Bacteria, College of Veterinary Medicine, South China Agricultural University, Guangzhou, China; ^3^Veterinary Diagnostic and Production Animal Medicine, College of Veterinary Medicine, Iowa State University, Ames, IA, United States

**Keywords:** *Campylobacter*, DNA repair, oxidative stress, antibiotic resistance, foodborne pathogens, endonuclease III

## Abstract

*Campylobacter jejuni* is a leading cause of foodborne illnesses worldwide. As a microaerobic pathogen, *C. jejuni* is subjected to DNA damages caused by various stresses such as reactive oxygen species (ROS) and UV radiations. The base excision repair (BER) system plays an important role in preventing mutations associated with oxidative DNA damage, but the system remains poorly characterized in *Campylobacter*. In this study, a BER homolog encoded by *cj0595c* (named *nth*) in *C. jejuni* was analyzed for endonuclease III activity and for its role in maintaining genomic stability. It was found that inactivation of *nth* resulted in elevated frequencies of spontaneous fluoroquinolone-resistant (FQ^R^) and oxidative stress resistant (OX^R^) mutants, compared with the wild-type strain in *C. jejuni*. Sequencing analysis of the FQ^R^ and OX^R^ mutants revealed that the elevated mutation rates were associated with C → T or G → A transition in *gyrA* (FQ^R^ mutants) *or perR* (for OX^R^ mutants). In an *in vitro* assay, a purified recombinant *C. jejuni* Nth protein demonstrated endonuclease III activity that recognized and excised the thymine glycol (Tg) base from a double stranded DNA. These findings indicate that Nth functions as a BER repair enzyme in *C. jejuni* and is important for the repair of DNA damage, protecting the bacteria from stresses encountered within a host and in the environment.

## Introduction

*Campylobacter jejuni*, a major bacterial foodborne pathogen, is among the most common causes of bacterial diarrhea in humans worldwide (Nachamkin et al., 2000). According to the United States Centers for Disease Control and Prevention (CDC), *C. jejuni* is estimated to cause 1.3 million infections and 120 deaths each year in the United States (Centers for Disease Control and Prevention [CDC], 2014). The main source of human *Campylobacter* infections is via contaminated food, water, or milk (Tauxe, 2002; Kassenborg et al., 2004). To survive in animal hosts and the environment, *C. jejuni* must be able to deal with various stresses such as reactive oxygen species (ROS) and UV radiations, which may lead to a variety of damages in the DNA structure, including single- or double-strand breaks, nucleotide modifications, abasic sites, bulky adducts, and mismatches (Eisen and Hanawalt, 1999). Bacteria have evolved sophisticated DNA repair mechanisms to defend against DNA damages and to retain their genetic integrity, including direct repair, excision repair and combinational repair, which have been well characterized in *Escherichia coli* and several other bacterial species (Eisen and Hanawalt, 1999; Friedberg, 2008). However, DNA repair mechanisms remain poorly defined in *Campylobacter*. It has been revealed through whole genome sequencing, that *C. jejuni* has an incomplete version of a DNA repair system compared to that found in *E. coli* (Parkhill et al., 2000; Gundogdu et al., 2007). It lacks important genes involved in DNA repair including *lexA*, which allows bacteria to mount a SOS response and survive sudden increases in DNA damage (Michel, 2005). MutH and MutL, which are methyl-directed mismatch repair (MMR) enzymes and repair replication errors that arise from mis-incorporations (mismatches) and strand slippage (frameshift errors) (Modrich, 1991), and MutM for base excision repair (BER), are also absent in *C. jejuni*.

In previous work, Gaasbeek et al. (2009) knocked out several putative DNA repair genes in *C. jejuni* including *mutS*, *uvrB*, *ung*, and *recA* and analyzed their effect on the emergence of spontaneous point mutations. None of the knocked-out mutants showed altered spontaneous point mutation frequency in *C. jejuni*. This is in contrast to findings in other bacteria where, mutations in the *mutS* loci have been associated with a hypermutability phenotype (Hsieh, 2001; Young and Ornston, 2001). Mutation of *ung* was also found to mediate increased mutation frequency in *Helicobacter pylori* (Huang et al., 2006), which is a close relative of *C. jejuni*. Therefore, the fact that inactivation of the known DNA repair genes did not change the spontaneous mutation rate in *Campylobacter*, suggests that there may be other mechanisms contributing to the repair of spontaneous point mutations in this organism. Indeed, our previous study revealed that MutY, an adenine glycosylase, which belongs to the BER system, plays an important role in the prevention of spontaneous point mutations, specifically the G → T or C → A transversion, in *C. jejuni* (Dai et al., 2015), indicating the BER system is functional in *C. jejuni*.

The BER system detects and repairs modified bases and plays an important role in preventing mutations associated with oxidative DNA damage such as 8-oxoguanine (David et al., 2007; Zharkov, 2008). The function of the BER system starts with the excision of the damaged bases by dedicated enzymes, namely DNA glycosylases (Zharkov, 2008). In *E. coli*, three major DNA glycosylases have been reported: the Uracil-DNA glycosylase superfamily, the Nth superfamily, and the Fpg/Nei superfamily (Zharkov, 2008). In *H. pylori*, strains lacking a functional endonuclease III (HpNth) showed slightly increased spontaneous mutation rates and were more susceptible than the parental strain to killing by exposure than to oxidative stress (O’Rourke et al., 2003). However, the function of the BER system has not been fully understood in *Campylobacter*. It remains unknown if BER enzymes other than MutY function in *Campylobacter*, affecting the spontaneous mutation rates and adaptating to endogenous or environmental stresses such as antimicrobials, oxidative stress and UV irradiation.

The goal of this study was to investigate the presence and functionality of BER homologs in *C. jejuni*. Using various methods, we found that the *nth* gene (*cj0595c*) encodes Endonuclease III of the BER system and inactivation of *nth* increased the spontaneous mutation frequencies with the C:G → T:A transition change, thus promoting the emergence of fluoroquinolone resistant (FQ^R^) and oxidative stress resistant (OX^R^) mutants in *C. jejuni*. Furthermore, it was found that a purified *C. jejuni* Nth protein was able to recognize and excise an oxidative damaged base from a double stranded DNA *in vitro*. The work identifies a new functional BER protein in *C. jejuni*, providing new insights into the DNA repair and adaptation mechanisms in this organism.

## Materials and Methods

### Identification of Putative BER Homologs in *C. jejuni*

The genome sequence of *C. jejuni* NCTC 11168 was searched for homologs of major BER enzymes of *E. coli* (Krwawicz et al., 2007; Zharkov, 2008) using the Basic local alignment search tool (BLAST)^1^. The similarities between the *C. jejuni* BER homologs and their *E. coli* equivalents were evaluated according to the query coverage and maximum identity values provided by the BLAST programs after alignment.

### Bacterial Strains and Growth Conditions

Bacterial strains used in this study are listed in Table 1. *C. jejuni* was cultured using Mueller-Hinton (MH) broth or agar (Difco) at 42°C under microaerobic conditions in jars filled with premixed gasses (5% O_2_, 10% CO_2,_ and 85% N_2_). *E. coli* was grown on Luria-Bertani (LB) agar or in an LB broth at 37°C for 24 h, under aerobic conditions.

**Table 1:** Bacterial strains used in this study.

Bacterial strain	^a^Relevant genotype or phenotype	Source
NCTC 11168	*C. jejuni* WT isolate	Parkhill et al., 2000
81–176	*C. jejuni* WT isolate	Hofreuter et al., 2006
IA3902	*C. jejuni* WT isolate	Sahin et al., 2008
Δ*nth*	11168 derivative; Δ*cj0595c*::*cat* insertional mutation	This study
Δ*nth*Com	Δ*nth* complement; Δ*cj0595c*::*cat* 16S:: *cj0595c*-*aphA3*	This study
81–176Δ*nth*	81–176 derivative; Δ*cj0595c*::*cat* insertional mutation	This study
IA3902Δ*nth*	IA3902 derivative; Δ*cj0595c*::*cat* insertional mutation	This study
Δ*cj1083c*	11168 derivative; Δ*cj1083c*::*aphA3* insertional mutation	This study
11168Δ*mutY*Δ*nth*	NCTC 11168 derivative; *mutY*::*cat*/Δ*cj0595c*::*aphA3*; Cm^R^/Km^R^	This study
81–176Δ*mutY*Δ*nth*	81–176 derivative; *mutY*::*cat*/Δ*cj0595c*::*aphA3*; Cm^R^/Km^R^	This study
3902Δ*mutY*Δ*nth*	3902 derivative; *mutY*::*cat*/Δ*cj0595c*::*aphA3*; Cm^R^/Km^R^	This study
Δ*mutY*	11168 derivative; Δ*mutY::cat* insertional mutation	Dai et al., 2015
81–176Δ*mutY*	81–176 derivative; Δ*mutY*::*cat* insertional mutation	This study
IA3902Δ*mutY*	IA3902 derivative; Δ*mutY*::*cat* insertional mutation	This study
Δ*uvrA*	11168 derivative; Δ*uvrA*::*cat* insertional mutation	This study
Δ*uvrC*	11168 derivative; Δ*uvrC*::*cat* insertional mutation	This study
11168 P*_katA_*-*cat*	11168 fusion construct; 11168 *16S*::P*_katA_-cat*	This study
Δ*nth* P*_katA_*-*cat*	Δ*nth* fusion construct; Δ*cj0595c*::*aac(3)IV 16S*::P*_katA_*-*cat*	This study


*^a^16S, 16S rRNA gene.*

### Construction of *C. jejuni* Knockout Mutants

The putative BER genes (*nth* and *cj1083c*) and two Nucleotide Excision Repair (NER) genes [*uvrA* (*cj0342c*) and *uvrC* (*cj1246c*)] of *C. jejuni* NCTC 11168 were knocked out by insertional mutagenesis. The primers used for gene inactivation are listed in Table 2. To construct a Δ*nth* strain, primers *nth*-5F and *nth*-5R were used to amplify a 506 bp fragment containing 5′ part of *nth* and its upstream region (*nth*-5′ fragment), while primers *nth*-3F and *nth*-3R were used to amplify a 562 bp fragment containing 3′ part of *nth* and its downstream region (*nth*-3′ fragment) (Supplementary Figure S1). Primer pairs *aphA3*-F/*aphA3*-R or *cat*-F/*cat*-R were used to amplify the *aphA3* (1,209 bp) or *cat* gene (817 bp) from pMW10 or pRY112 encoding kanamycin or chloramphenicol resistance, respectively, using the Phusion High-Fidelity DNA Polymerase (New England Biolabs). After *Kpn*I and *BamH*I digestion, the *nth*-5′, *cat* and *nth*-3′ PCR fragments were ligated by the T4 DNA ligase (New England Biolabs) and PCR amplified utilizing *nth*-5F and *nth*-3R primers, resulting in the construction of the *nth*-5′-*cat*-*nth*-3′ PCR product. The purified *nth*-5′-*cat*-*nth*-3′ product was then electroporated into *C. jejuni* NCTC 11168. Transformants were selected on MH agar plates containing 10 mg/L of chloramphenicol. To construct a Δ*mutY*Δ*nth* double knockout strain, a similar *nth*-5′-*aphA3*-*nth* -3′ PCR product was constructed as described for Δ*nth* above. The purified *nth*-5′-*aphA3*-*nth*-3′ product was then electroporated into the *C. jejuni* Δ*mutY* strain, which was constructed in our previous study (Dai et al., 2015). Transformants were selected on MH agar plates containing both 30 mg/L kanamycin and 10 mg/L of chloramphenicol. The insertion of the resistance marker into the *nth* and *mutY* genes was confirmed by PCR using primers *nth*-5F/*nth*-3R (Table 2) and *mutY*-5F/*mutY*-3R (Dai et al., 2015). A similar strategy was used to knockout mutants Δcj*1083c*, Δ*uvrA*, and Δ*uvrC* in *C. jejuni* strains NCTC 11168, 81–176 and IA3902.

**Table 2:** PCR primers used in this study.

Primers	^a^Sequences	Restriction sites	PCR product size (bp)
*nth*-5F	CTTTAACTTTAGCCGCATC		506
*nth*-5R	**CGGGGTACC**AATTTAAGCTCTGTTACAGGC	*Kpn*I	
*nth*-3F	**CGCGGATCC**TAACTATCTTCATCAAGCCAT	*BamH*I	562
*nth*-3R	AATTTCTTTCTCTTTGTTCGAT		
*nth-*Fc	**CTAGTCTAGA**TTCTGTATCGCTAATGCTC	*Xba*I	881
*nth-*Rc	**CTAGTCTAGA**CACTAGGCTTGTAAGGTTC	*Xba*I	
*cj1083c*-5F	GCCTTAGCAGATATCATCG		502
*cj1083c*-5R	**CGGGGTACC**AGCTTGGTAAAAATTTGTGCTC	*Kpn*I	
*cj1083c*-3F	**CGCGGATCC**AGAAAATGCGAACTTTACGAA	*BamH*I	566
*cj1083c*-3R	AATATTAGGCGTAAGAATGTC		
*uvrA*-5F	GGACTTTTAATTGCTCCGAT	*EcoR*I	541
*uvrA-*5R	**CGGGGTACC**CTTCAGCATAAAGAGTTCCA	*Kpn*I	
*uvrA-*3F	**CGCGGATCC**CGCTTTATATACTTGATGAACCT	*BamH*I	665
*uvrA-*3R	AAAGAATATTTCGCAAAACCA		
*uvrC*-5F	GCCTTTATATCCGCAAGCA		685
*uvrC*-5R	**CGGGGTACC**AGCAAAATAAGATCTAACACGA	*Kpn*I	
*uvrC*-3F	**CGCGGATCC**AAGCTTGCAAATTTAGGAC	*BamH*I	542
*uvrC*-3R	CTTCATTTAAGCAACGCAT		
*aphA3*-F	**TATGGTACC**CGCTTATCAATATATCTATAGAATG	*Kpn*I	1209
*aphA3*-R	**CGCGGATCC**GATAATGCTAAGACAATCACTAAA	*BamH*I	
*cat*-F	**CGGTGGTACC**TGGAGCGGACAACGAGTAAA	*Kpn*I	817
*cat*-R	**CGCGGATCC**TCAGTGCGACAAACTGGGATT	*BamH*I	
nth-HisF	CACCATCACCATCACGGATCCAAAAGAAATTTAGAAATCAAAGAAC		624
nth-HisR	CCAAGCTCAGCTAATTAAGCTTTCATTTAAGTTCCTTATCTTTACTTTTG		
cj1083c-HisF	GGATCGCATCACCATCACCATCACGGATCCACTGGAGCACAAATTTTTAC		684
cj1083c-HisR	ACAGGAGTCCAAGCTCAGCTAATTAAGCTTTCATAAATCTTCCTTTAAAATTTTAATC		

*^a^The nucleotides in bold and underlined represent the restriction sites added to the primers (KpnI, BamHI, or XbaI) with three additional bases at the 5′ end to facilitate enzyme recognition and cleavage as suggested by the manufacturer (New England BioLabs, United States).*

### Complementation of the *C. jejuni* Mutant Strains

The Δ*nth* mutant was complemented by inserting a wild-type copy of the *nth* gene between the 16S and 23S rRNAs as described by Muraoka and Zhang (2011). The primers used are listed in Table 2. Briefly, primers *nth-*Fc and *nth*-Rc were used to amplify the intact *nth* gene including its promoter. The amplicon was digested with *Xba*I and cloned into the pRRK plasmid, which contains an *aphA3* cassette in the opposite orientation to the ribosomal genes, to obtain plasmid construct pRRK*-nth*. The direction of the insertion was confirmed by primers *nth*-Fc and *aphA3*-F/*aphA3*-R. The construct with *nth* in the same transcriptional direction as the ribosomal genes was selected and used as the suicide vector to insert the *nth* gene into the chromosome of the Δ*nth* strain. The complemented strain named Δ*nth*Com was selected on MH agar containing 30 μg/ml of kanamycin and was confirmed by PCR using primers *nth*-Fc and *nth*-Rc.

### Assay of Spontaneous FQ^R^ Mutation Frequencies

To determine the spontaneous FQ^R^ mutation frequencies in *Campylobacter*, the methods of Bjorkholm et al. (2001) and Hanninen and Hannula (2007) were used with minor modifications. Briefly, bacterial strains (FQ-susceptible) were grown in 3 ml of MH broth for 24 h, after which 30 μl aliquots (∼10^5^ CFU) of each strain were distributed into 20 tubes containing 3 ml of MH broth. Cultures were incubated with shaking for 24 h at 42°C. After incubation, colony counts (CFU) of evolved mutants in each tube were determined by spreading 1.5 ml on MH agar plates containing 1 μg/mL ciprofloxacin (10 × MIC). The number of total viable bacteria was determined from three tubes by dropping 10 μl of 10^−4^, 10^−5^, and 10^−6^ dilutions on non-selective MH agar plates. After 2–4 days of incubation in a microaerobic atmosphere at 42°C, colonies were counted. The frequency of resistant mutants was expressed as the median number of resistant colonies divided by the mean of the total number of viable cells. The mutation frequencies were calculated from the median of 20 cultures for the respective strain. Thus, the occasional jackpot cultures had no impact on the calculation and the obtained values (Bjorkholm et al., 2001). Several colonies growing on selective MH plates from the spontaneous FQ^R^ mutation frequency test were randomly picked to sequence the QRDR region in the *gyrA* gene. To ensure that the 30 μl of bacterial cultures used to inoculate the 3 ml broth samples were free of any preexisting resistant mutants, the same volume (30 μl) was also plated on a selective plate. The mutation frequency of each strain was measured only if no preexisting mutants were present in the 30 μl aliquots used as inoculum.

### Determination of Spontaneous OX^R^ Mutation Frequencies

It was not possible to determine the spontaneous OX^R^ mutation frequency directly by using H_2_O_2_ as a selection agent, since this compound is not stable and tends to degrade rapidly in the culture medium. Therefore, a reporter system was developed by fusing the promoter of *katA* (an oxidative stress response gene) with the chloramphenicol resistance gene *cat*, generating *P_katA_-cat* gene (Dai et al., 2017). This method was utilized in this study to compare spontaneous OX^R^ mutation frequencies of the wild-type and the Δ*nth* mutant strains. Since the fusion gene P_*katA*_-*cat* plasmid construct harbors both *aphA3* and *cat* resistance cassettes, an additional resistance cassette *aac(3)IV*, which contributes to apramycin resistance, was utilized to construct a new *nth* mutant in the 11168 isolate as described in a previous report (Cameron and Gaynor, 2014), generating Δ*nth*::*aac(3)IV*. The P_*katA*_-*cat* gene was then inserted into the genome of *C. jejuni* NCTC 11168 and Δ*nth*::*aac(3)IV* isolates, generating 11168 P*_katA_*-*cat* and Δ*nth* P*_katA_*-*cat* constructs. Similar to *C. jejuni* NCTC 11168, both constructs were susceptible to chloramphenicol (MIC = 1–2 μg/mL) as transcription of the P*_katA_*-*cat* gene was inhibited by the oxidative stress regulator PerR. However, spontaneous *perR* mutations, which compromise the PerR function, would lead to the derepression of the *katA* promoter of P*_katA_*-*cat* and consequently elevate the MIC of chloramphenicol (16–32 μg/mL) of the PerR mutants from both 11168 P*_katA_*-*cat* and Δ*nth* P*_katA_*-*cat* (Dai et al., 2017). Therefore, MH agar plates containing 6 μg/mL of chloramphenicol were used in this study to measure spontaneous *perR* loss-of-function mutations in *C. jejuni* 11168 P*_katA_*-*cat* and Δ*nth* P*_katA_*-*cat*. The spontaneous chloramphenicol resistance (Cm^R^) mutation frequencies were determined as previously described for enumerating spontaneous ciprofloxacin and ampicillin resistance mutations in *C. jejuni* (Dai et al., 2015). Colonies growing on selective MH plates from the spontaneous Cm^R^ mutation frequency test were randomly picked to sequence the *perR* gene. In addition, these colonies were sub-cultured and tested for their susceptibility to H_2_O_2_ using disk diffusion assay as described below.

### Oxidant Susceptibility Assay

A disk inhibition assay was utilized to compare the sensitivity to oxidants between *C. jejuni* WT and the OX^R^ mutants as described previously (Palyada et al., 2009). Each *C. jejuni* strain was tested for susceptibility to 3% H_2_O_2_, 3% cumene hydroperoxide in DMSO, and 100 mM paraquat dichloride in H_2_O, respectively.

### Assay of Susceptibility to UV Induced DNA Damage

Previous studies found that the disruption of the Nucleotide Excision Repair (NER) gene *uvrB* resulted in a significant reduction in viability after exposure to UV irradiation in *C. jejuni*, due to a lack of repair of bulky DNA damages induced by UV irradiation (Gaasbeek et al., 2009). To determine if *C. jejuni* BER genes were involved in the repair of bulky DNA damages, such as pyrimidine dimers induced by UV irradiation, *C. jejuni* wild-type 11168 and several DNA repair gene mutants were tested for susceptibility to UV irradiation. The methods of Gaasbeek et al. (2009) was utilized with minor changes. Overnight *C. jejuni* cultures grown on MH agar plates were suspended in MH broth to an OD_600_ of 0.2. Two hundred μl of each suspension was added into a 24-well cell culture plate (3 replicates/isolate), which was then placed in a UV cross-linker and exposed to UV irradiation at 312 nm. The irradiation was done with a density of 0.12 J cm^−2^ for 1 min. Then, serial dilutions (10^0^–10^6^) of UV-exposed and non-exposed cells were dropped on MH plates for enumeration of viable cells. After 24–48 h of incubation, the numbers of colonies were counted. All tests were performed in triplicates. The results were analyzed using a one-way ANOVA followed by a Dunnett’s test using the GraphPad Prism software.

### Expression and Purification of rNth and rCj1083c From *E. coli*

The BER enzyme Nth in *E. coli* is known for its ability to remove damaged pyrimidines from double-stranded DNA, especially the Thymine glycol (Tg) (Saito et al., 1997), which is induced by oxidative damage and has been shown to be a lethal lesion *in vitro* and *in vivo* (Hariharan et al., 1977; Clark and Beardsley, 1986). To investigate if the Nth and Cj1083c proteins in *C. jejuni* repairs oxidative DNA damages, full-length histidine-tagged recombinant Nth (rNth) and Cj1083c (rCj1083c) of *C. jejuni* were produced in *E. coli* JM109 strain by using the pQE-30 vector (Qiagen). The complete coding sequences of *nth* and *cj1083c* gene in the isolates were amplified with primers nth-HisF/nth-HisR and cj1083c-HisF/cj1083c-HisR (Table 2). The amplified PCR products were ligated into the pQE-30 vector, which had been previously digested with *Bam*HI and *Hind*III, utilizing the SLiCE cloning method (Zhang et al., 2012). The plasmid in the *E. coli* clones producing the Nth or Cj1083c proteins were sequenced, confirming the cloned constructs. *E. coli* harboring pQE-30-*nth* or pQE-30-*cj1083c* were grown in LB broth at 37°C with shaking at 250 rpm to an OD_600_ of 0.4–0.6. The expression of recombinant Nth and Cj1083c were induced by addition of 0.4 mM IPTG for 5 h at 28°C with shaking at 200 rpm. Purification of the rNth and Cj1083c proteins were performed following the procedures described previously (Zhang et al., 2000; Lin et al., 2002). The purified rNth and Cj1083c were desalted using PD-10 Desalting Columns (GE Healthcare Life Sciences, United States) and then stocked in 50% glycerol solution containing 20 mM Tris, pH 7.4, 100 mM NaCl, 1 mM EDTA, and 1 mM 1,4-Dithiothreitol (DTT) at −20°C.

### Oligonucleotide Substrate Preparation for Endonuclease III Activity Assay

A previously reported 30-mer oligonucleotide substrate oligo30-F-Tg, containing a Tg residue at position 13 (GATCCTCTAGAG**Tg**CGACCTGCAGGCATGCA) (Marenstein et al., 2003), was synthesized and PAGE purified by Integrated DNA Technologies (United States). The 30-mer oligonucleotide oligo30-F containing a Thymine (T) of the identical sequence and the complementary oligonucleotide oligo30-R containing a mismatch base Guanine (G) against Tg or T were also synthesized. Nucleotides were annealed to the complementary strand according to the manufacturer’s instructions, which generated two double-strand DNA fragments nth-Tg-DNA and nth-control-DNA, containing the Tg:G or T:G pairs, respectively. These two DNA fragments were stocked at −20°C for subsequent endonuclease III activity assay.

### Endonuclease III Activity Assay

Purified rNth and rCj1083c of *C. jejuni* were assayed for endonuclease III activity. A commercially available Nth from *E. coli* (New England Biolabs) was included as a positive control. An rPerR protein (Dai et al., 2017), which has no endonuclease III activity and was produced and purified from the same *E. coli* host with the same methods as for rNth and rCj1083c in this study, was used as a negative control. The endonuclease III activity assay was carried out following the manufacturer’s instructions. Briefly, 1 pmol of nth-Tg-DNA or nth-control-DNA was treated with either 1 μl (10 U) *E. coli* Nth, or *C. jejuni* rNth (2 pmol), rCj1083c (2 pmol) and rPerR (2 pmol) for overnight at 37°C in 1 X NEB reaction buffer containing 1 mM DTT, 20 mM Tris-HCl, and 1 mM EDTA (pH 8.0). The reaction mixture (10 μl) was then mixed with an equal volume of Gel loading Buffer II (Thermo Fisher Scientific, United States), followed by heating at 95°C for 5 min to denature any secondary DNA structure to generate single stranded DNA. Samples were then separated by 15% Denaturing urea polyacrylamide gel electrophoresis (Urea PAGE) in 7 M urea and 1 × Tris borate-EDTA (TBE) buffer at 200 V for 1 h. The Urea PAGE gel was stained using SYBR Gold Nucleic Acid Gel Stain (Thermo Fisher Scientific, United States). Gel images were taken with a digital imaging system under UV light at 254 nm.

## Results

### Identification of Putative BER Genes in *C. jejuni*

BLAST search of the *C. jejuni* NCTC11168 genome for BER genes did not identify any *fpg* or *nei* homologs in the genome. Searching for annotations of DNA glycosylases in *C. jejuni* revealed that *cj0086c*, *nth*, and *cj1620c* genes are likely to encode *C. jejuni* Uracil-DNA glycosylase (Ung), endonuclease III (Nth), and adenine DNA glycosylase (MutY), respectively (Table 3). Interestingly, the *cj1083c* gene was also predicted and annotated as an endonuclease III encoding gene in the NCTC 11168 genome, even though its protein sequence shares a relatively lower homology (30%) to Nth in *E. coli*. Further alignment of amino acid sequences of *E. coli* endonuclease III, *C. jejuni* Nth, and *C. jejuni* Cj1083c by PROMALS3D (Xia et al., 2009) revealed that all three proteins contain conserved alpha-helix secondary structures (Supplementary Figure S2). Deduced from the crystal structure of *E. coli* endonuclease III (Thayer et al., 1995), *C. jejuni* Nth and Cj1083c harbor a relatively conserved Helix-hairpin-helix (HhH) motif that may be involved in DNA binding (Supplementary Figure S2). However, amino acids that are predicted to be important for Nth functions in *E. coli* are less conserved in *C. jejuni* Cj1083c (Supplementary Figure S2). For example, Asp120 in *E. coli* Nth, which is important for the DNA lyase activity, was only found in *C. jejuni* Nth (Asp118) but not at the corresponding site in Cj1083c (Glu126). Gln41 in *E. coli* Nth, which may be important for DNA binding, is also conserved in *C. jejuni* Nth (Gln39) but not in Cj1083c (Asn42). Furthermore, the alignment showed that *C. jejuni* Cj1083c lacks an iron/sulfur cluster region, which was proposed to have a structural role for the DNA repair (Guan et al., 1998; Johnson et al., 2005). Among these genes, *cj0086c* (*ung*) and *cj1620c* (*mutY*) has been functionally characterized in *Campylobacter* (Gaasbeek et al., 2009; Dai et al., 2015). However, the role of *nth* and *cj1083c* in DNA repair has not been determined and is thus investigated in this study.

**Table 3:** BER gene orthologs identified in *C. jejuni* NCTC 11168 genome in comparison with those in *E. coli* K-12.

*E. coli* str. K-12 substr. MG1655		*C. jejuni* subsp. *jejuni* NCTC 11168		^a^Similarities (%)	
Gene	Locus tag	Annotation	Locus tag	Identities	Positivities
*ung*	b2580	Uracil-DNA-glycosylase	cj0086c	47	66
*nth*	b1633	Endonuclease III	cj0595c	40	57
*nth*	b1633	Endonuclease III	cj1083c	30	42
*mutY*	B2961	Adenine DNA glycosylase	cj1620c	35	53

*^a^The similarities (at amino acid level) were determined by BLAST programs after alignment.*

### Elevated Spontaneous FQ^R^ Mutation Frequency in the Δ*nth* Mutant but Not in the Δ*cj1083c* Mutant

The *nth* and *cj1083c* genes were knocked out to determine their impact on frequencies of spontaneous ciprofloxacin resistant mutants (due to point mutations in the *gyrA* gene) (Table 4). Compared to the wild-type 11168, disruption of *cj1083c* did not cause any change in the FQ^R^ mutation frequency. However, inactivation of *nth* resulted in 3 to 10-fold (*p* < 0.05, *t*-test) increase in the frequencies of FQ^R^ mutants in three different *C. jejuni* strains including 11168, IA3902 and 81–176 (Table 4). Complementation of Δ*nth* with an intact copy of the *nth* gene from the 11168 strain, restored the spontaneous FQ^R^ mutation frequencies to the wild type level (Table 4). Interestingly, the *C. jejuni* double knockout strain Δ*mutY*Δ*nth* exhibited a further increase of the FQ^R^ mutation frequency compared with the Δ*nth* strain (Table 4), but the increase was comparable to that from the MutY mutant alone, as reported in the previous study (Dai et al., 2015), suggesting that the *mutY* effect is dominant, which overshadows the impact of the *nth* mutation on the spontaneous FQ^R^ mutation frequencies in *Campylobacter*. Several FQ^R^ colonies, grown on selective plates, were picked from the Δ*nth* background and subsequently sequenced for the QRDR region in the *gyrA* gene. The mutants harbored a C257 → T (Thr-86-Ile) substitution or a G268 → A (Asp-90-Asn) transition and had MIC values between 8 and 16 mg/L. Altogether, the results indicated that the Δ*nth* mutation, but not Δ*cj1083c* mutation, increased the spontaneous FQ^R^ mutation frequency in *C. jejuni*.

**Table 4:** Spontaneous FQ^R^ mutation frequencies of different *C. jejuni* strains.

	Spontaneous FQ^R^ frequencies		
*C. jejuni* Strains	NCTC 11168	IA3902	81–176
Wild type	1.6 × 10^−9^	0.65 × 10^−9^	2.3 × 10^−9^
Δ*cj1083c*	1.1 × 10^−9^	^a^NT	NT
Δ*nth*	^b^1.4 × 10^−8^	^b^5.1 × 10^−9^	^b^6.8 × 10^−9^
Δ*nth*Com	^c^1.2 × 10^−9^	NT	NT
Δ*mutY*	0.61 × 10^−7^	0.72 × 10^−8^	1.8 × 10^−7^
Δ*mutY*Δ*nth*	^d^1.2 × 10^−7^	^d^1.4 × 10^−8^	^d^2.4 × 10^−7^

*^a^NT, not tested. ^b^Statistically significant difference (p < 0.05) by t-test between Δnth and its respective wild type strains. ^c^Statistically significant difference (p < 0.05) by t-test between ΔnthCom and Δnth. ^d^Statistically significant difference (p < 0.05) by t-test between ΔmutYΔnth and Δnth strains.*

### Elevated Spontaneous OX^R^ Mutation Frequency in the Δ*nth* Mutant

To further examine the role of *nth* in DNA repair, we determined its effect on the spontaneous OX^R^ frequency, which is due to spontaneous loss-of-function mutations in the peroxide regulator *perR* in *C. jejuni* (Dai et al., 2017). Previously, we used a promoter-reporter fusion gene P*_katA_*-*cat* to determine the spontaneous OX^R^ mutation frequencies in *C. jejuni* (Dai et al., 2017). The fusion gene *P_katA_-cat* was then inserted into the genome of *C. jejuni* 11168 and Δ*nth* strains, generating 11168 *P_katA_-cat* and Δ*nth P_katA_-cat* strains. The spontaneous chloramphenicol resistance (Cm^R^) mutation frequencies were determined by plating on chloramphenicol-containing plates. The average Cm^R^ mutation frequency for the Δ*nth* P*_katA_*-*cat* isolate was 1.1 × 10^−7^, ∼6-fold higher than that of the *C. jejuni* 11168 P*_katA_*-*cat* isolate (1.9 × 10^−8^) (*P* < 0.05; *t*-test). Colonies grown on chloramphenicol plates were randomly picked from both 11168 P*_katA_*-*cat* or Δ*nth* P*_katA_*-*cat* and subsequently sequenced for the *perR* gene. All sequenced Cm^R^ colonies carried mutations in the *perR* gene (Table 5). For strain 11168 P*_katA_*-*cat*, no unique mutation pattern was observed. However, all colonies sequenced from Δ*nth* P*_katA_*-*cat* carried a C → T or G → A transition in the PerR encoding sequence. These transition mutations led to amino acid (aa) changes or formation of a stop codon in PerR, which caused early termination of PerR translation. In addition, four Cm^R^ colonies from the Δ*nth* P*_katA_*-*cat* carried the same C250T mutation causing aa change at 84His in PerR, which is the metal binding site and essential for the regulatory function of PerR (Pohl et al., 2003; Lee and Helmann, 2007; Lucarelli et al., 2007). The result indicated that the Cm^R^ mutants generated from the Δ*nth* strain had distinct *perR* mutation patterns, which are G:C → A:T transitions. This is consistent with the result found in *E. coli*, for which a previous study showed that mutation of Nth caused G:C → A:T transitions due to lack of repair of the pyrimidine oxidative damages in the *E. coli* mutants (Blaisdell et al., 1999). The spontaneous Cm^R^ mutants of both 11168 P*_katA_*-*cat* and Δ*nth* P*_katA_*-*cat* strains were also assayed for susceptibility to different oxidants by a disk inhibition assay. As shown in Table 6, the Cm^R^ colonies were highly resistant to the oxidant treatments. Representative pictures of the disk inhibition results are shown in Supplementary Figure S3. This result is consistent with what we found in our previous work that the Cm^R^ phenotype corresponded with the OX^R^ phenotype in *C. jejuni* strain carrying the fusion gene P*_katA_*-*cat* (Dai et al., 2017). Altogether, these findings indicate that the Δ*nth* mutation increased the loss-of-function mutations (C → T or G → A transition) in *perR*, leading to a PerR malfunction and a subsequent derepression of the PerR-controlled genes, which in turn led to the Cm^R^ and OX^R^ phenotypes in *C. jejuni*.

**Table 5:** *perR* mutations identified in the spontaneous OX^R^ mutants from *C. jejuni* 11168 P*_katA_*-*cat* and Δ*nth* P*_katA_*-*cat* isolates.

11168 P*_katA_*-*cat*		Δ*nth* P*_katA_*-*cat*	
Mutation^a^	AA change	Mutation^a^	AA change
−A (36)	Frameshift	G266A	89Cys → Tyr
C88T	30His → Tyr	C184T	62Gln → Stop codon
G266A	89Cys → Tyr	G395A	132Cys → Tyr
−A (75)	Frameshift	C250T	84His → Tyr
C91G	31Pro → Ala	C250T	84His → Tyr
−T (130)	Frameshift	C250T	84His → Tyr
C149T	50Ala → Val	C178T	60Gln → Stop codon
C28G	10His → Asp	G266A	89Cys → Tyr
		C250T	84His → Tyr
		C217T	73Gln → Stop codon

*^a^The numbers are in relative to the first nucleotide sequence of the perR gene or the first amino acid in the PerR protein based on C. jejuni NCTC 1168 genome. “−” in front of a nucleotide indicates a base deletion.*

**Table 6:** Oxidative stress sensitivity of *C. jejuni* strains and constructs as measured by a disk diffusion assay.

Strain	^a^Mean diam (mm) of zone of inhibition		
	H_2_O_2_	Cumene hydroperoxide	Paraquat dichloride
NCTC11168	22.5	32	11.5
11168 P*_katA_*-*cat* OX^R^ mutant	^b^6	^b^24	^b^6
Δ*nth* P*_katA_*-*cat* OX^R^ mutant	^b^6	^b^25.5	^b^6

*^a^The data are expressed as the diameters (in millimeters) of the zones of inhibition. Data are shown as the means of triplicate plates. The diameter of the disk itself is 6 mm, and therefore 6 mm indicates no obvious inhibition. ^b^Statistically significant difference (p < 0.05) by t-test was observed between the mutant and wild type strains.*

### *C. jejuni* Nth but Not Cj1083c Recognizes and Excises Thymine Glycol (Tg) Base From Double Stranded DNA

**FIGURE 1:**
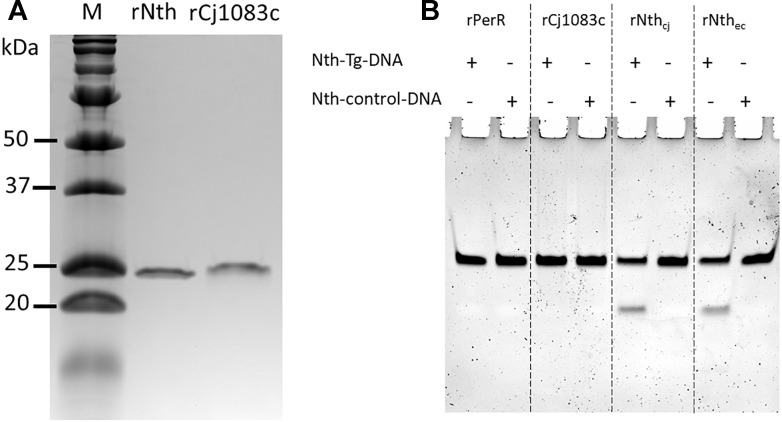
Production of the *C. jejuni* rNth and rCj1083c proteins in *E. coli* and evaluation of their Endonuclease III activities *in vitro*. **(A)** SDS-PAGE analysis of *C. jejuni* rNth and rCj1083c expressed in *E. coli*. Lane M contains molecular mass markers (Bio-Rad), while lane rNth or rCj1083c shows rNth or rCj1083c of *C. jejuni* NCTC 11168 purified by Ni-nitrilotriacetic acid affinity chromatography, respectively. **(B)** Urea PAGE analysis of endonuclease III activities of *C. jejuni* rPerR, rCj1083c, rNth (rNth_cj_) and *E. coli* Nth (rNth_ec_), proteins. The ‘+’ and “–” signs indicate the presence and absence of the assayed DNA, respectively.

rNth and rCj1083c were successfully purified from *E. coli* with predicted molecular weights 25.03 and 27.79 kDa, respectively (Figure 1A). Figure 1B shows the activity of *C. jejuni* rPerR, rCj1083c, rNth, and *E. coli* Nth on two 30 bp double-strand DNA fragments: nth-Tg-DNA and nth-control-DNA, which contained the Tg:G or T:G pairs, respectively. As expected, *E. coli* Nth showed obvious endonuclease III activity on the nth-Tg-DNA fragment, which was shown by a cleaved fragment visible on the gel. Similarly, the *C. jejuni* rNth protein also exhibited endonuclease III activity on the nth-Tg-DNA fragment (Figure 1B). No cleaved product was observed for the nth-control-DNA fragment after treatment with either *E. coli* Nth or *C. jejuni* rNth. The negative control (rPerR) and rCj1083c did not yield a visible cleaved fragment on either of the nth-Tg-DNA or nth-control-DNA, suggesting that rCj1083c may not function as an endonuclease III in *C. jejuni*. The above results indicate that the *C. jejuni* Nth protein, instead of Cj1083c, recognizes and excises Tg base in the Tg:G pair instead of the T:G mismatch pair. The results also suggest that Nth is important for the repair of oxidative DNA damages in *Campylobacter*.

### The BER Genes in *C. jejuni* Are Not Involved in the Bulky DNA Damage Repair

**FIGURE 2:**
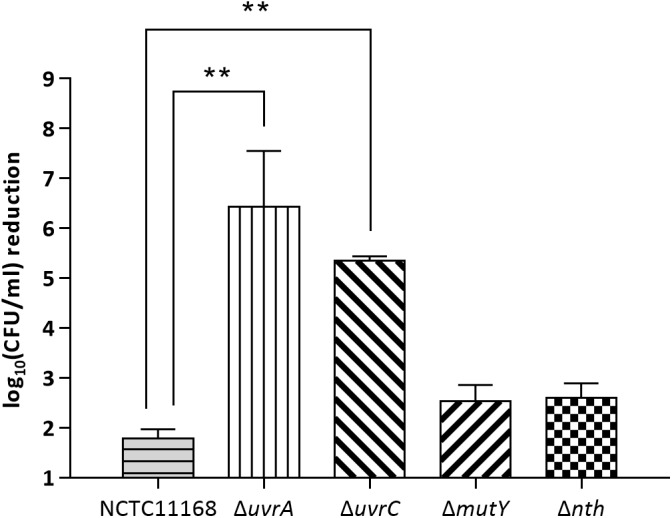
UV-mediated reduction of cell viability in *C. jejuni* NCTC 11168 and various mutant strains. The bars indicate standard errors of triplicate experiments. Statistically significant differences from the wild-type NCTC 11168 are marked by double asterisks (^∗∗^) (*p* < 0.01).

To evaluate if the BER genes are also involved in repairing bulky DNA damages, *mutY* and *nth* were assayed for their effect on susceptibility of *C. jejuni* to UV irradiation in this study. Two NER genes *uvrA* and *uvrC*, which encode a multienzyme complex UvrABC with *uvrB* gene, were included as positive controls in this study. As shown in Figure 2, knockout of either the *uvrA* or *uvrC* gene caused 10^4^–10^6^-fold reductions in viability compared to the wild type strain after UV irradiation (*p* < 0.05). However, the Δ*mutY*, Δ*nth* mutant strains did not show a significant difference (*p* > 0.05) in viability compared to that of the *C. jejuni* 11168 wild type strain. These results indicate that in *C. jejuni*, the BER genes *mutY and nth* are not involved in repairing DNA damages caused by UV irradiation.

## Discussion

Although the genome of *C. jejuni* harbors homologs of the excision repair systems, including NER and BER (Parkhill et al., 2000; Gaasbeek et al., 2009), most of them remain functionally uncharacterized. In this study two BER homologs, *nth* (*cj0595c*) and *cj1083c*, in *C. jejuni* were analyzed. It was revealed that inactivation of *nth*, but not *cj1083c*, increased FQ^R^ and OX^R^ mutation frequencies, which were attributable to mutations in either the *gyrA* gene (related to the FQ^R^ phenotype) or the *perR* gene (related to the OX^R^ phenotype). Notably, all of the spontaneous point mutations in the *gyrA* or *perR* genes of FQ^R^ or OX^R^ mutants from the Δ*nth* background are G:C → A:T transitions, which are regarded as a signature mutation pattern for Nth deficiency in *E. coli* (Blaisdell et al., 1999). Endonuclease III activity assay further revealed that the purified recombinant Nth protein recognized and excised Tg containing double-stranded DNA (Figure 1B). Altogether, these results indicate that Cj0595c functions as an Endonuclease III and is an Nth ortholog that is important for DNA damage repair in *Campylobacter*.

In other bacteria, the endonuclease III (Nth) protein, together with endonuclease VIII (Nei), is responsible for repairing oxidized pyrimidines including Tg, which would cause G:C → A:T transitions in *E. coli* (Wang et al., 1998; Blaisdell et al., 1999). However, there are no endonuclease VIII homologs in the *C. jejuni* genome (Parkhill et al., 2000). Our results showed that inactivation of Nth elevated the mutation rate with the C → T or G → A transition in *Campylobacter*. This was evident from the observation that all the FQ^R^ mutants of the Nth mutant strain carried C257 → T or G268 → A transition in the QRDR region of *gyrA* and that all the spontaneous OX^R^ mutants of the Nth mutant strain carried a C → T or G → A transition in the *perR* gene (Table 5). These results suggest that Nth functions effectively in *Campylobacter* despite the absence of a Nei homolog. Whether there are other DNA repair enzymes involved in the repair of oxidized pyrimidines in *Campylobacter* remains to be investigated in further studies.

We previously reported that the mutation of the BER protein MutY caused a >100 fold increase in spontaneous FQ^R^ and OX^R^ mutation frequencies in *C. jejuni* (Dai et al., 2015, 2017), which is much higher than the increase of mutation frequencies caused by the Nth mutation (3–10-fold) as observed in the current study (Table 4). Similar results were also observed with *E. coli* and *H. pylori*, in which the Nth mutant only showed a mild mutator phenotype (Gifford and Wallace, 2000; O’Rourke et al., 2003). The difference between MutY and Nth in their impact on spontaneous mutation frequencies could be explained by the fact that they repair different types of mutations. Regardless, the successful complementation of the Δ*nth* strain, with an intact *nth* gene to the wild-type level mutation frequency and the uniformed mutation patterns (G:C → A:T transitions) observed in the spontaneous FQ^R^ and OX^R^ mutants of the Δ*nth* mutant strains, clearly indicate that the increase in the spontaneous mutation frequencies is due to the Nth mutation in *C. jejuni*.

The mutations caused by MutY deficiency are predominantly G:C → T:A transversions in *C. jejuni* (Dai et al., 2015, 2017). This is due to the fact that *mutY* encodes an adenine glycosylase that specifically repairs the G:C → T:A transversions, which are caused by adenine/7,8-dihydro-8-oxoguanine (8-oxoG) mismatches (Cheng et al., 1992; Michaels and Miller, 1992). Tg and 8-oxoG are two of the most common DNA lesions that result from ROS or ionizing radiation (Basu et al., 1989; Kanvah et al., 2010). The ability of Nth and MutY to repair these DNA lesions, associated with oxidative damage, suggests that the BER system plays an important role in repairing oxidative DNA damages in *Campylobacter*, facilitating its adaptation in the host and environment.

Both the previous study (Dai et al., 2015) and the current work clearly demonstrates that Nth and MutY are responsible for fixing point mutations in *Campylobacter*. However, neither Nth nor MutY deficiency affected *C. jejuni* susceptibility to UV irradiation, which generates bulky DNA damage. On the contrary, inactivation of *uvrA* or *uvrC* significantly reduced the viability of *C. jejuni* after UV irradiation (10^4^–10^6^ fold) (Figure 2). This is consistent with a previous finding that *uvrB* mutation in *C. jejuni* drastically decreased the cell viability after a brief UV irradiation (Gaasbeek et al., 2009). These results suggest that *C. jejuni* requires the UvrABC system to repair UV induced bulky DNA damages. Thus, the BER and NER pathways in *C. jejuni* appear to have distinct and complementary functions, with the former functioning to repair single base changes and the latter fixing bulky DNA damages such as pyrimidine dimers that affect overall DNA structure.

Although Cj1083c is also annotated as endonuclease III in the genome of *C. jejuni* NCTC 11168 and shares 30% aa homology with the Nth in *E. coli*, mutation of this gene did not alter the spontaneous mutation frequencies in *C. jejuni*. Additionally, an *in vitro* enzymatic assay confirmed that rCj1083c lacks endonuclease III activity (Figure 1B). This is also consistent with the bioinformatics analysis (Supplementary Figure S2), which revealed that Cj1083c lacks an iron/sulfur cluster region important for DNA binding in *E. coli* Nth (Thayer et al., 1995). Although Cj1083c shares a general alpha-helix secondary structure with *E. coli* Nth and *C. jejuni* Nth, the key amino acids involved in DNA binding are less conserved. These observations suggest that Cj1083c may have a different function other than that of an endonuclease III. In *H. pylori*, the Cj1083c homolog, HP0602 (44% aa identify), was found to encode a novel 3-Methyladenine DNA Glycosylase (MagIII) within the Endonuclease III family (O’Rourke et al., 2000). MagIII was found to be able to recognize and release 3-methyladenine, instead of oxidized bases from modified DNA. Whether Cj1083c in *C. jejuni* has a similar function than a DNA repair enzyme in *H. pylori* requires further investigation.

In summary, the findings of the current study reveals a previously undefined Nth-like protein (encoded by *cj0595c*) involved in DNA repair in *C. jejuni*. This protein functions as Endonuclease III and repairs damaged pyrimidines in DNA. The presence of functional BER repair mechanisms, including MutY and Nth, protects *C. jejuni* cells from oxidative DNA damages encountered frequently within a host and in the environment.

## Author Contributions

LD and QZ contributed conception and design of the study. LD wrote the first draft of the manuscript. LD, JX, OS, and QZ contributed to manuscript revision, read and approved the submitted version.

## Conflict of Interest Statement

The authors declare that the research was conducted in the absence of any commercial or financial relationships that could be construed as a potential conflict of interest.

## Footnotes

1 http://blast.ncbi.nlm.nih.gov/Blast.cgi
