# Supplementary material for: Identification of a nth-Like Gene Encoding an Endonuclease III in Campylobacter jejuni
Source: Front Microbiol. 2019 Apr 10;10:698. doi: 10.3389/fmicb.2019.00698 (PMC6467930; doi:10.3389/fmicb.2019.00698)
Supplement: Supplementary file 1 [file Table_1.DOCX]

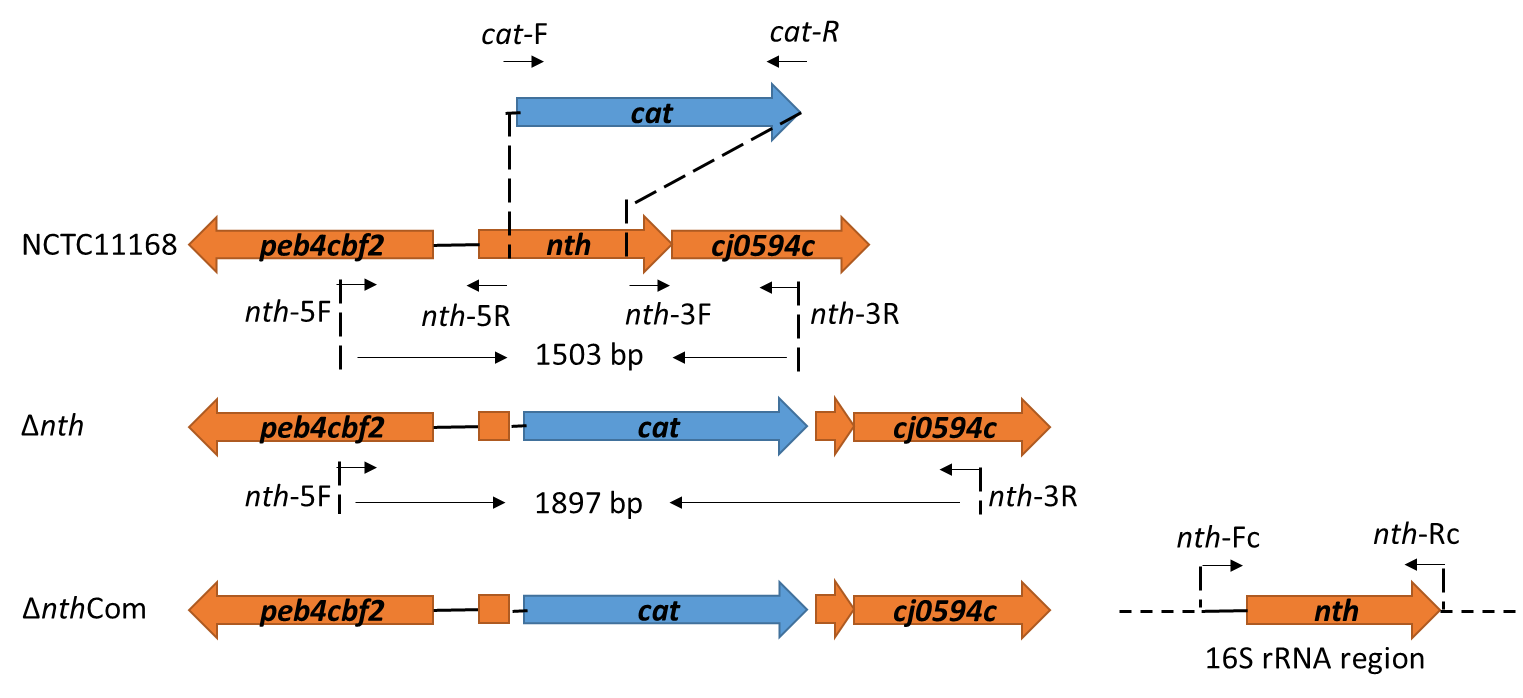

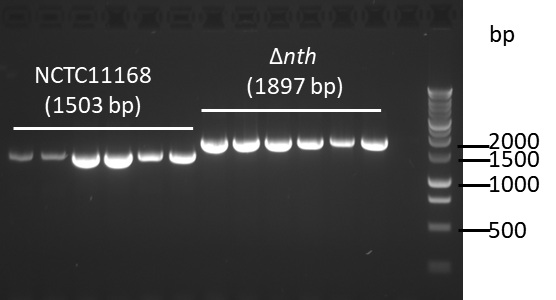
 A B

Figure S1. Strategies for the construction of the *nth* deletion mutant. (A) The schematic diagram of the genomic structure of *nth* and its flanking region in *C. jejuni* wild type strain NCTC11168, *nth* mutant (Δ*nth*), and the complemented construct (Δ*nth*Com). The block arrows represent different genes and their directions of transcription in the *C. jejuni* strains. The small arrows represent the locations and directions of the specific primers. The “16S rRNA region” represent a distant genomic location where an extra copy of an intact *nth* gene with its own promoter was inserted. The numbers (in bp) between two long arrows with vertical dashed lines display the sizes of the PCR products for the confirmation of the *nth* mutant construct. (B) A representative gel image showing PCR confirmation of the *nth* mutant construct.


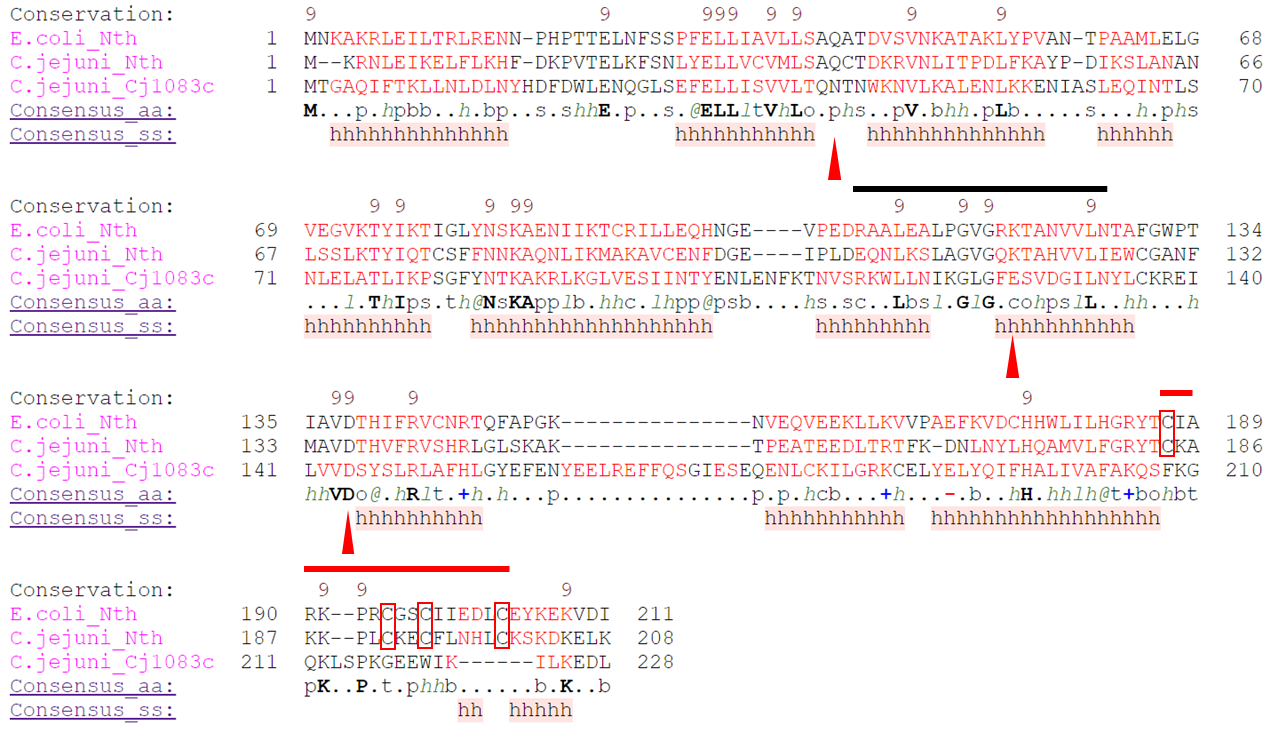
Figure S2. The colored alignment results of *E. coli* Nth, *C. jejuni* Nth and Cj1083c proteins using PROMALS3D web server. The first line in each block shows conservation indices for positions with a conservation index of 9 (identical amino acids). Sequences which were colored in red represent a predicted secondary structure of alpha-helix in the protein. The fourth line from the top with different characters represents the consensus amino acid symbols: conserved amino acids are in bold and uppercase letters; aliphatic (I, V, L): *l*; aromatic (Y, H, W, F): *@*; hydrophobic (W, F, Y, M, L, I, V, A, C, T, H): h; alcohol (S, T): o; polar residues (D, E, H, K, N, Q, R, S, T): p; tiny (A, G, C, S): t; small (A, G, C, S, V, N, D, T, P): s; bulky residues (E, F, I, K, L, M, Q, R, W, Y): b; positively charged (K, R, H): **+**; negatively charged (D, E): **-**; charged (D, E, K, R, H): c. The symbol “h” on the last line in each block represent a consensus predicted alpha-helix secondary structure. The sequence region under a black line represents the potential DNA binding site with an HhH helix motif. Amino acids which are important for *E. coli* Nth functions are highlighted by the red arrow heads. The sequence region under a red line represents an iron/sulfur cluster with four representative cysteine residues (not present in the Cj1083c protein) highlighted by red squares.


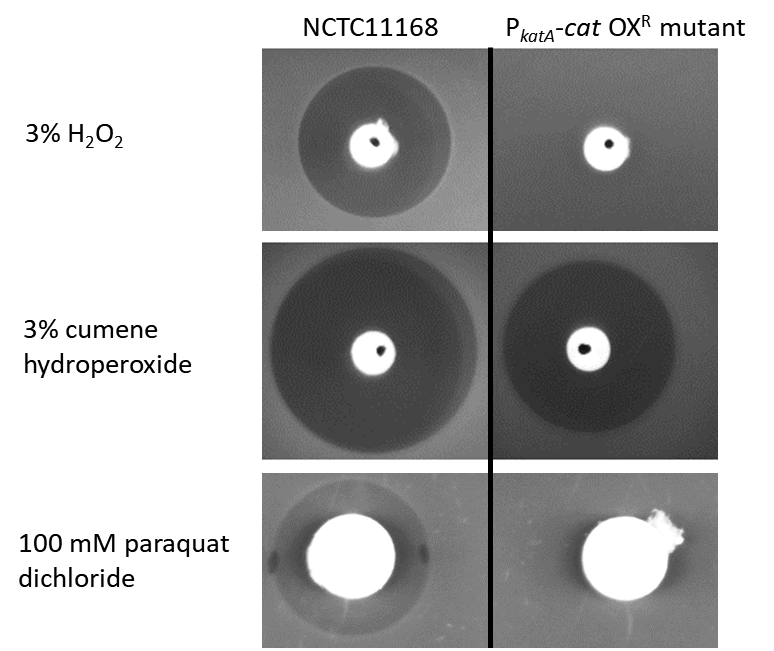
Figure S3. Representative pictures of the disk inhibition assay of two *C. jejuni* NCTC11168 and the P*_katA_*-*cat* OX^R^ mutant using three different oxidants. The images on the left represent the results for *C. jejuni* NCTC11168. The images on the right represent the results for the P*_katA_*-*cat* OX^R^ mutant.
